# Sex Differences in Frailty in Milan Over the Last 2000 Years: A Hazards‐Based and Cumulative Phenotype Approach

**DOI:** 10.1002/ajpa.70111

**Published:** 2025-08-13

**Authors:** Lucie Biehler‐Gomez, Kathryn E. Marklein, Samantha L. Yaussy, Douglas E. Crews, Sharon N. DeWitte, Cristina Cattaneo

**Affiliations:** ^1^ Laboratory of Forensic Anthropology and Odontology (LABANOF), Department of Biomedical Sciences for Health University of Milan Milan Italy; ^2^ Department of Anthropology University of Louisville Louisville Kentucky USA; ^3^ Center for Archaeology and Cultural Heritage University of Louisville Louisville Kentucky USA; ^4^ Department of Sociology and Anthropology James Madison University Harrisonburg Virginia USA; ^5^ Department of Anthropology The Ohio State University Columbus Ohio USA; ^6^ Institute of Behavioral Science Department of Anthropology University of Colorado Boulder Boulder Colorado USA

**Keywords:** bioarchaeology, cumulative stress, frailty, hazards analysis, stress markers

## Abstract

**Objectives:**

Frailty in bioarchaeology has garnered increasing interest in recent decades, particularly for analyzing and comparing past health across different groups and populations. A hazards‐based cumulative phenotype approach was applied to 492 adult males and females from five consecutive historical periods in the city of Milan: Roman (2nd–5th century CE), Early Middle Ages (6th–10th century CE), Late Middle Ages (11th–15th century CE), Modern (16th–18th century CE), and Contemporary (20th century CE).

**Materials and Methods:**

After estimating mortality and survivorship risks (hazards and survival analyses) individually associated with 10 biomarkers, population‐specific frailty indices were constructed to explore differences in frailty between sexes and periods.

**Results:**

When all periods were considered, a 4‐biomarker frailty index was constructed: the presence of *cribra orbitalia*, *cribra femoralis/humeralis*, porotic hyperostosis, and osteoarthritis (absence) is associated with higher mortality. Lower survivorship (Kaplan–Meier) and higher risk of mortality (Cox proportional hazards) were associated with higher frailty index values. Significantly higher frailty values were observed in the Late Middle Ages, correlating with a general worsening of living conditions in the Middle Ages, and the lowest frailty was observed in Contemporary individuals. Comparisons of 4‐biomarker frailty indices between sexes revealed no significant differences overall or by period.

**Discussion:**

As females are biologically buffered, their cumulative frailty should be lower than males; comparable cumulative frailty suggests cultural factors may be impacting female frailty. This study contributes to methodological advancements in bioarchaeological frailty analysis and provides insights into the trends of health in Milan over the past 2000 years.

## 1 Introduction

Frailty has long been a topic of interest in bioarchaeological research, either as an explicit focus of research or as a complicating factor in interpretative analyses. Much of the research examining biosocial variation in health or frailty over the last 30 years has been influenced by Wood et al.'s (1992) paper detailing the eponymous “Osteological Paradox”. Among the most pressing concerns encompassed by the Osteological Paradox are the effects that heterogeneous frailty and selective mortality have on the composition of bioarchaeological assemblages of human remains and thus on our inferences regarding the health of once‐living populations. Heterogeneous frailty refers to variation among individuals in their susceptibility to disease and death relative to individuals of the same age. This heterogeneity is shaped by a variety of biological, environmental, and social factors, such as genetics, epigenetics, exposures to developmental stressors, wealth inequality, and social marginalization. Because of heterogeneous frailty, mortality at each age tends to be selective (non‐random) such that people with the highest frailty at each age are most likely to die at that age and thus become part of the death assemblages that bioarchaeologists study. The inherent bias of skeletal remains toward the least healthy people at each age is further complicated by the fact that skeletal indicators of stress or disease are not perfectly specific or sensitive measures of health or stress exposures. For many scholars, the most salient point made by Wood and colleagues is the possibility that the presence and, particularly, absence of skeletal biomarkers are more complex than had conventionally been viewed. The presence of skeletal biomarkers tells us something concrete, at the very least, about an individual's exposure to a stressor—i.e., at the most basic level, a skeletal biomarker indicates that some insult (often among many possible etiologies) caused a disruption in homeostasis. However, the absence of a skeletal biomarker may plausibly reflect diametrically opposed scenarios: an individual so frail that they died upon exposure to a stressor without manifesting an observable skeletal response, or an individual never exposed to such stressors (and thus with low frailty as a result of non‐exposure), or with low inherent frailty such that they efficiently responded to a stressor without manifesting a relatively severe, skeletal response. Wood and colleagues' detailed discussion of the idea that the presence (or absence) of skeletal biomarkers of infectious disease, malnutrition, or other conditions is not necessarily directly reflective of underlying health or frailty has motivated a reorientation in how many scholars approach the topic.

Bioarchaeologists interested in directly studying or accounting for frailty in investigations of health, and particularly health disparities or the social determinants of health in the past, have operationalized the phenomenon in various ways, heeding Wood and colleagues' call to consider the various sources of bias that may shape observable patterns of skeletal biomarkers in human skeletal remains. We highlight two major approaches here: hazards‐based and cumulative phenotype approaches. Hazards‐based approaches explicitly or implicitly recognize that survival or hazards of death reflect important underlying levels of health at the individual and aggregate population level. Simply put, long lives reflect good general health (or low frailty), and short lives reflect the opposite. This is arguably an oversimplification (e.g., it does not account for the myriad ways that health might be defined for past or living people). However, it has utility in bioarchaeological research, allowing us to potentially infer meaningful information about variation in health in the past using inherently biased data from human skeletal remains. Hazards‐based approaches integrate the definition of frailty provided by Vaupel et al. (1979) and Wood et al. (1992): risk of death relative to age peers. Complementarily, cumulative phenotype approaches operationalize skeletal frailty as an index of stressors that accumulate during life and are observable on skeletal remains. Among the living, frailty indices reflecting functional losses are common components of research and practice across anthropology, gerontology, medicine, and physiology (Fried et al. 2004; Fried et al. 2001). Among both skeletal and living samples, frailty reflects a cumulative outcome from multiple and repeated stressor exposures resulting in accumulated physical and physiological damage and losses and shows synergistic influences with comorbid conditions and likely allostatic load (Fried et al. 2004; Gruenewald et al. 2009; Jeszka et al. 2024; Szanton et al. 2009).

Typically, cumulative phenotype and hazards‐based approaches to studying frailty have been applied independently. However, recognizing the potential complementary nature of these different conceptualizations and operationalizations of frailty, these approaches were previously integrated to produce novel, context‐specific indices of frailty and resilience incorporating multiple skeletal biomarkers and informed by hazards and survival analysis (Yaussy et al. 2024). This approach allowed implementation of risk of death (or conversely survival) as the ultimate measure of frailty and accounted for the potential effects of accumulated stress exposures and comorbidities, while also attending to the possibility that exposures and outcomes may be highly historically‐ and culturally‐contingent, i.e., local biologies (Niewöhner and Lock 2018).

Milan has a long history as a dynamic and influential city. Its origins date to 590 bce when it was founded by the Celts. Following conquest by Rome in 222 bce, it became known as *Mediolanum* (Calderini 1953). The city occupies a strategic geographic and political location, situated on fertile lands near rivers and at the intersection of major trade routes connecting eastern and western Italy as well as northern Europe to Rome, benefitting from abundant natural resources and socioeconomic activity. In Antiquity, the city was capital of the Western Roman Empire and maintained its political, cultural, and economic centrality well into the Middle Ages (Santos Salazar 2021; Violante 1953).

From the Early Middle Ages (6th–10th centuries) onward, Milan particularly developed its healthcare system, with institutions such as the *xenodochia*—early forms of hospitals—providing aid to the impoverished and needy (Albini 2016). In the late 13th century, Bonvesin da la Riva's work, *De magnalibus Mediolani—Meraviglie di Milano*, praised the city's prosperity, listing its abundant water resources, temperate climate, skilled artisans, and fertile lands producing diverse crops (da la Riva 1997). He also applauded Milan's numerous hospitals, noting 10 hospitals within the city that cared for the “poor and sick”. These facilities provided not only shelter and food but also medical and surgical care, demonstrating a strong tradition of public welfare. Throughout the Middle Ages, and even more markedly during the Modern era (16th–18th centuries), Milan's political prominence fostered private and public charity, serving as a significant force against poverty and contributing to an improved standard of living for all, including the underprivileged (Albini 2016; Biehler‐Gomez, del Bo, et al. 2023).

A major milestone in Milan's healthcare history occurred in 1456 ce, when Duke Francesco Sforza established the *Ospedale Maggiore*, known affectionately by the Milanese as the *Ca' Granda* (from “Casa Grande” or “Big Factory”). This initiative was part of broader social and sanitary reforms and aimed to centralize the administration of all existing hospitals in the city. The *Ospedale Maggiore* became a model for healthcare innovation and scientific advancement across Europe, focusing on treating the impoverished in acute need (Mattia et al. 2022). Throughout the Modern and Contemporary (19th–20th centuries) periods, the hospital's role extended beyond medical care; it helped mitigate social unrest by providing essential support to the city's poor, including abandoned mothers and children (Reggiani 2014).

Bioarchaeological research has shed light on the health trends of historic Milanese populations. Previous studies evidenced continuity in stature of adult males and females over the past 2000 years (Biehler‐Gomez, del Bo, et al. 2023), alongside a higher frequency of individuals with multiple traumata (i.e., presence of more than one trauma in different body regions (Redfern 2017)), notably adult male individuals, during the Middle Ages compared to other periods (Biehler‐Gomez, Moro, et al. 2023). Regarding frailty, analysis using the Global History of Health Project Index, Skeletal Frailty Index, and Biological Index of Frailty (Petrosino et al. 2025) revealed, in a smaller population sample, a gradual increase from the Roman era (2nd–5th centuries CE) to the Late Middle Ages (11th–15th centuries CE), followed by a decline into the Contemporary era. Kaplan–Meier survival analyses, along with Cox proportional and Gompertz hazard models (Biehler‐Gomez et al. 2024b), demonstrated an upward trend in female longevity from the Roman to Contemporary eras, with a plateau during the Middle Ages and Modern period. Females also showed higher mortality risks and lower survivorship in the Roman and Modern eras with respect to their male counterparts, but this pattern reversed in the Contemporary period. Further examination using biomarkers as covariates (Biehler‐Gomez, Yaussy, et al. 2025) showed that *cribra orbitalia*, *cribra femoralis* and/or *humeralis*, porotic hyperostosis, and linear enamel hypoplasia correlated with decreased survival, contrary to osteoarthrosis and Schmorl's nodes. Conversely, markers like Harris lines and antemortem trauma had no significant impact on lifespan. The relatively low frequency of these markers in Roman females suggested that other factors, such as childbirth, likely contributed to female frailty. In contrast, during the Modern era, post‐cranial cribriotic lesions were associated with decreased longevity in females, suggesting heightened exposure to environmental stressors, while this was observed in the Roman, Early Middle Ages, and Modern eras for males (Biehler‐Gomez, Yaussy, et al. 2025).

The current paper integrates hazards‐based and cumulative phenotype (indexical) approaches to generate a frailty index specific to the Milanese biocultural and historical context. Given previously identified disparities in risks of mortality and survivorship between estimated females and estimated males in Milan, we anticipate higher frailty index values among females compared to males in each historical period.

## 2 Studied Individuals

The study sample consists of 492 individuals from the Anthropological Collection of the Laboratory of Forensic Anthropology and Odontology (*Collezione Antropologica LABANOF*) curated at the University of Milan (Cattaneo et al. 2018). The collection is one of the largest in the world, with over 10,000 skeletons from the city of Milan and across Lombardy. Established in 2017, it was officially recognized as an osteological museum collection by the Lombardy region in 2018.

The 492 individuals analyzed in this study originated from eight archaeological sites in Milan and represent almost 2000 years of Milanese history (Biehler‐Gomez et al. 2024b): excavations under the current *Università Cattolica*, dated to the Roman period (2nd–5th century CE); the Ambrosian basilica of *San Dionigi* (5th century CE); excavations at *Sant'Ambrogio* Basilica, spanning Roman period (1st–2nd century CE) to Late Middle Ages (15th century CE); *San Vittore*, with burials dated to the Roman period (3rd–4th century CE) through the Modern age (16th–17th century CE). Additionally, the excavation at Via *Necchi* revealed stratigraphy extending from the Roman period through to the Late Middle Ages, while mass graves at *Viale Sabotino*, likely resulting from the Manzoni plague, date to the mid‐17th century CE. The sample also includes remains from the *Ca' Granda* hospital (17th century CE) and the CAL Milano Cemetery Skeletal Collection, a modern osteological assemblage of unclaimed individuals who died in the second half of the 20th century, in accordance with Italian regulations.

All individuals are associated with urban Milanese contexts and—apart from the Contemporary skeletal remains for whom dates of birth and death are available—have been dated using a combination of stratigraphic analysis, associated artifacts, and radiocarbon dating. They have been categorized into five historical periods, as per previous studies (Biehler‐Gomez, Yaussy, et al. 2025; Biehler‐Gomez et al. 2024b): 104 individuals (59 females and 45 males) from the Roman Era (1st–5th century CE), 89 individuals (47 females and 42 males) from the Early Middle Ages (6th–10th century CE), 121 individuals (56 females and 65 males) from the Late Middle Ages (11th–15th century CE), 91 individuals (43 females and 48 males) from the Modern Era (16th–18th century CE), and 87 individuals (48 females and 39 males) from the Contemporary Era (19th–20th century CE). Only individuals with fused ossa coxae (i.e., fused ilium, pubis, and ischium) were selected for sex estimation to be reliably assessed. The archaeological context, including the topography of the burial sites, cultural material found with the remains, and burial structures, suggests that the individuals likely belonged to the lower or middle socioeconomic strata of Milanese society. This consistent socioeconomic background across the sites provides a basis for more uniform diachronic analysis. Additionally, the urban setting of the necropolises offers a relatively stable geographic and historical context, minimizing social and economic disparities within the sample (Biehler‐Gomez, Yaussy, et al. 2025; Biehler‐Gomez et al. 2024b).

### 2.1 Ethics Statement

This research is part of the wider DOMINA project (*Donne Milanesi Nascoste* or Hidden Women of Milan), which focuses on exploring the experiences of women in Milan over the past 2000 years through the analysis of their skeletal remains (Biehler‐Gomez et al. 2024a). The deceased individuals analyzed in the current study are drawn from multiple historical contexts and research efforts within the project (Biehler‐Gomez, del Bo, et al. 2023; Biehler‐Gomez, Gibelli, et al. 2025; Biehler‐Gomez et al. 2022; Biehler‐Gomez, Moro, et al. 2025, 2023; Biehler‐Gomez, Yaussy, et al. 2025; Biehler‐Gomez et al. 2024b). The privilege to study these individuals is granted through Article 43 of the Mortuary Police Regulations (D.P.R. September 10, 1990, 285) (Cattaneo et al. 2018), and research projects therein reflect collaborations between the *Soprintendenze Archeologia Belle Arti e Paesaggio* of Lombardy, the Municipality of Milan, and the LABANOF. The analysis of the archaeological remains was conducted under agreement with the *Sopraintendenza Archeologia, Belle Arti e Paesaggio della Lombardia*, the regional authority for cultural heritage within the Italian Ministry of Cultural Heritage. This research adhered to the ethical and scientific guidelines established by the agreement. The examination of the anonymized contemporary remains was carried out in compliance with Article 43 of Presidential Decree No. 285, dated September 10, 1990, of the National Police Mortuary Regulation and following the protocols set with the Health Territorial Agency of the city of Milan. As such, informed consent was not required. All procedures were performed in accordance with Italian legislation, as well as institutional standards and regulations. In addition to the academic potential DOMINA promises, since its inception in 2020, researchers have actively engaged with the local Milanese community, fostering public support through ongoing publications and outreach efforts^1^. This engagement reflects the community's growing interest in uncovering and preserving Milan's biocultural heritage.

## 3 Methods

Osteological analyses were conducted to estimate biological sex, age‐at‐death, stature, and for pathological and trauma analysis. Biological sex was estimated from morphology and expression of features on the pelvis and cranium (Phenice 1969; Walker 2005, 2008), supplemented by metric analyses (Spradley and Jantz 2011). Age‐at‐death was estimated using a variety of methods, including dental eruption (AlQahtani et al. 2010), epiphyseal closure (Cunningham et al. 2016), and age‐related changes to the pubic symphysis (Brooks and Suchey 1990), auricular surface (Buckberry and Chamberlain 2002; Lovejoy et al. 1985), acetabulum (Rougé‐Maillart et al. 2009), and sternal end of the fourth rib (Iscan and Loth 1986). Age estimates were then grouped into the following categories: 16–20, 21–30, 31–45, 46–60, 61–80, and over 80 years. In total, 10 biomarkers were considered for the present study: eight were included as per previous research (Biehler‐Gomez, Yaussy, et al. 2025), namely *cribra orbitalia* (CO), *cribra femoralis* and/or *humeralis* (CF/CH), porotic hyperostosis (PH), linear enamel hypoplasia (LEH), Harris lines (HL), osteoarthrosis (OA), Schmorl's nodes (SN), and antemortem trauma (AM), and two more were added for the purpose of this study, including stunted stature (SS) and periosteal new bone formation (PNBF). Following previous studies (DeWitte 2014; Yaussy et al. 2024), we use data on PNBF from the tibia. We selected the tibia as it is a robust bone and thus tends to be relatively well preserved in archaeological contexts. Further, we focus on PNBF on just one element because increasing the number of elements risks reducing the number of individuals who can be included in the study if there is intra‐ and inter‐individual variation in the preservation of skeletal elements (as is often the case in bioarchaeology). *Cribra femoralis* and *cribra humeralis* were pooled together (i.e., considered present if at least one of the two lesions was observed) to maximize the possibilities of recognizing a trend. Regarding stature, regression equations were used on fused long bones, selecting, in order of priority, the femur or tibia when present. Descriptions and scoring of the biomarkers are outlined in Table 1.

**TABLE 1:** Skeletal and dental biomarkers of stress included in this study with references, conditions of observability, and scoring.

Biomarker	Description	References	Conditions of observability	Scoring
*Cribra orbitalia* (CO)	Porotic lesion on the orbital roof	(Brickley 2018)	One orbital roof well‐preserved	Present/absent
*Cribra femoralis* and/or *humeralis* (CF/CH)	Porotic lesion on the neck of the femur (*femoralis*) and/or below the head of the humerus (*humeralis*)	(Brickley 2018; Schats 2021)	One neck of femur or humerus well‐preserved	Present/absent
Porotic hyperostosis (PH)	Porotic lesion on the cranial vault	(Brickley 2018; Stuart‐Macadam 1985)	Occipital and/or posterior parietal bones well‐preserved	Present/absent
Linear enamel hypoplasia (LEH)	Linear horizontal defects on the tooth enamel surface	(Goodman and Armelagos 1985; Goodman and Rose 1990)	One monoradicular tooth well‐preserved	Present/absent
Harris lines (HL)	Transverse radiopaque lines on x‐rays of long bones	(Harris 1931)	Radiographs of at least one long bone	Present/absent
Stunted stature (SS)	Stature, calculated based on long bone lengths using the formulae by Trotter (1970), two standard deviations below the mean per sex and period group	(DeWitte and Hughes‐Morey 2012)	One long bone preserved allowing for stature estimation	Present/absent
Periosteal new bone formation (PNBF)	Bone deposition on the cortical surface of the tibia (plaque‐like, irregular elevation)	(Rana et al. 2009; Weston 2008)	Cortical surface of one tibial shaft preserved	Present/absent
Osteoarthrosis (OA)	Degenerative joint disease manifesting as marginal and central osteophytes, eburnation, pitting, and alteration of the articular surface	(Rogers et al. 1987; Waldron 2008)	One weight‐bearing articulation preserved	Present/absent
Schmorl's nodes (SN)	Erosive lesions on the vertebral body from intervertebral disk herniation	(Dar et al. 2010; Kyere et al. 2012)	One thoracic or lumbar vertebral body	Present/absent
Antemortem trauma (AM)	Bone discontinuity (fracture, microfracture), dislocations, and/or signs of fracture healing (callus)	(Lovell 1997)	None	Present/absent

A significant limitation to the original skeletal frailty index model in bioarchaeology is the assumption that all skeletal biomarkers equally reflect frailty (Marklein et al. 2016). This assumption fails to recognize the variability that exists in skeletal lesions, as well as human biological variation and the influence of cultural context on skeletal lesions and frailty. As Wood et al. (1992) remind paleopathologists, the presence or absence of skeletal lesions has the potential to reflect resilience, as well as frailty, in a given population. For example, given that osteoarthrosis (OA) is typically found in higher frequencies among older adults, previous studies of survivorship and risk of death in Milan (Biehler‐Gomez et al. 2024b) and London (Yaussy et al. 2024) have suggested it is most appropriately viewed as a marker of resilience, rather than frailty. In contrast, linear enamel hypoplasia (LEH) is often found to coincide with higher risks of death and reduced longevity (Armelagos et al. 2009; Boldsen 2007; Yaussy et al. 2024), which suggests it is a particularly robust indicator of frailty.

To better discern which biomarkers were indicative of frailty and which biomarkers might be applied and combined into a context‐specific frailty index, survival analysis and hazards analysis were applied to each of the 10 biomarkers listed above. Kaplan–Meier survival analysis was used to assess the associations between survivorship and each of the biomarkers, and a log‐rank test was used to identify significant (*p* < 0.10) differences in survivorship based on the presence or absence of each biomarker.

Hazards analysis (Cox proportional hazards model and Gompertz mortality model) was used to examine the associations between hazards of death and the presence of each biomarker. The semiparametric Cox proportional hazards model does not require the specification of the baseline hazard function and tests the null hypothesis that the covariate (i.e., the presence of a given biomarker) has no effect on the hazard. The reported hazard ratio indicates the change in risk of death associated with a unit increase in the covariate, and significant hazard ratios (*p* < 0.10) that are greater than 1.0 indicate that the presence of the biomarker is associated with increased risk of death. All Kaplan–Meier survival and Cox proportional hazards analyses were conducted in SPSS version 29 (IBM Corp 2023).

The fully parametric Gompertz model of adult mortality is a biomathematical hazard model that reflects the age‐related physiological processes that influence mortality (Gompertz 1825). The Gompertz mortality function includes two parameters that describe the relatively low risk of death characteristic of young adult ages and the increasing risk of death associated with senescence (Wood et al. 2002). In this stage of the analysis, the presence of each biomarker was modeled as a covariate affecting the Gompertz model using a proportional hazard specification:

$$h_{i}\left(t_{i}|x_{i}\rho \right)=h\left(t_{i}\right)e^{\left(x_{i}\rho \right)}$$

where the baseline Gompertz hazard *h(t*_*i*_ *) = αe* ^𝞫*t*^, *t*_*i*_ is the age of the *i*th skeleton in years, *x*_*i*_ is the biomarker covariate, and *ρ* is the parameter representing the effect of the covariate on the baseline hazard. A positive estimate for the parameter representing the effect of the covariate would suggest individuals who exhibited a given biomarker were at an increased risk of death compared to individuals who did not exhibit that biomarker. The Gompertz model parameters were estimated using maximum likelihood analysis with the program *mle* (Holman 2005). A likelihood ratio test (LRT) was then used to compare the fits of the full model (i.e., which includes the biomarker covariate) and the baseline model (i.e., which does not include the biomarker covariate). The LRT tests the null hypothesis that there is no difference in risk of mortality between individuals who do and do not exhibit the biomarker (*H*_*0*_: effect of biomarker covariate = 0). The LRT was computed as follows: LRT = −2[ln(*L*_*biomarker*_)–ln(*L*_*baseline*_)], where LRT approximates a *χ* ^*2*^ distribution with df = 1. For the Gompertz analyses, an alpha level of 0.10 was selected to indicate statistical significance.

Although *p* values are used as a guide to interpret the results of this study, we recognize that concerns have been raised by scholars regarding the use of *p* values to identify significant results in medical and epidemiological research (e.g., Cohen 2011; Lang et al. 1998; Rothman 1998; Trafimow and Marks 2015). Particularly in bioarchaeological studies, small sample sizes may affect the likelihood of identifying “significant” results (according to a traditional alpha level of 0.05), despite consistent and noteworthy patterns in the data. Therefore, for each of the analyses described above, a *p* value of less than 0.10 is considered indicative of a trend and worthy of consideration.

The results of Kaplan–Meier analyses for the presence or absence of individual biomarkers are shown in Table 2. Some of these results were published in a recent paper emphasizing the impacts of each biomarker on survivorship across sex categories and historical periods (Biehler‐Gomez, Yaussy, et al. 2025). The presence of CO, CF/CH, PH, LEH, and PNBF is each associated with significantly lower survivorship. The presence of OA and SN is associated with significantly higher survivorship. There are no significant associations between survivorship and the presence of HL, SS, or AM.

**TABLE 2:** Kaplan–Meier survival analysis of presence versus absence of individual biomarkers (*n* = number of individuals with the lesion).

Biomarker	Presence versus absence	Mean age at death (95% CI)	*p*
*Cribra orbitalia* (CO) *n* = 290	Presence *n* = 73	40.541 (37.194–43.887)	< 0.001***
	Absence *n* = 217	49.074 (46.570–51.578)	
*Cribra femoralis* and/or *humeralis* (CF/CH) *n* = 393	Presence *n* = 69	34.362 (31.036–37.689)	< 0.001***
	Absence *n* = 324	46.836 (44.936–48.737)	
Porotic hyperostosis (PH) *n* = 313	Presence *n* = 34	35.500 (30.919–40.081)	< 0.001***
	Absence *n* = 279	47.423 (45.293–49.553)	
Linear enamel hypoplasia (LEH) *n* = 250	Presence *n* = 127	39.094 (36.299–41.890)	0.069*
	Absence *n* = 123	43.130 (40.544–45.716)	
Harris lines (HL) *n* = 387	Presence *n* = 108	44.056 (41.036–47.075)	0.303
	Absence *n* = 279	45.609 (43.487–47.731)	
Stunted stature (SS) *n* = 356	Presence *n* = 26	46.290 (44.395–48.184)	0.794
	Absence *n* = 330	43.222 (26.584–59.861)	
Periosteal new bone formation (PNBF) *n* = 371	Presence *n* = 91	43.269 (40.270–46.268)	0.078*
	Absence *n* = 280	46.352 (44.095–48.609)	
Osteoarthrosis (OA) *n* = 431	Presence *n* = 210	53.790 (51.616–55.965)	< 0.001***
	Absence *n* = 221	35.557 (33.695–37.419)	
Schmorl's nodes (SN) *n* = 386	Presence *n* = 180	46.961 (44.419–49.503)	0.096*
	Absence *n* = 206	43.121 (40.624–45.619)	
Antemortem trauma (AM) *n* = 435	Presence *n* = 163	46.080 (43.476–48.684)	0.199
	Absence *n* = 272	43.353 (41.217–45.489)	

*Note:* Mean survival times (mean ages at death) in years are shown with 95% confidence intervals in parentheses (*p < 0.10, **p < 0.05, ***p < 0.001).

The results of Cox proportional hazards analyses for presence or absence of individual biomarkers are presented in Table 3. The presence of CO, CF/CH, and PH are each associated with significantly higher hazards of death, whereas the presence of OA is associated with significantly lower hazards of death. There are no significant associations between hazard of death and the presence of LEH, HL, SS, PNBF, SN, or AM.

**TABLE 3:** Cox proportional hazards analysis of presence versus absence of individual biomarkers.

Biomarker	Exp(*β*) (95% CI)	*p*
*Cribra orbitalia* (CO) *n* = 290	1.540 (1.173–2.020)	0.002**
*Cribra femoralis* and/or *humeralis* (CF/CH) *n* = 393	1.849 (1.418–2.412)	< 0.001***
Porotic hyperostosis (PH) *n* = 313	1.805 (1.256–2.594)	< 0.001***
Linear enamel hypoplasia (LEH) *n* = 250	1.177 (0.918–1.508)	0.199
Harris lines (HL) *n* = 387	1.091 (0.873–1.363)	0.444
Stunted stature (SS) *n* = 356	1.064 (0.548–2.066)	0.854
Periosteal new bone formation (PNBF) *n* = 371	1.179 (0.927–1.498)	0.179
Osteoarthrosis (OA) *n* = 431	0.435 (0.356–0.531)	< 0.001***
Schmorl's nodes (SN) *n* = 386	0.882 (0.722–1.078)	0.219
Antemortem trauma (AM) *n* = 435	0.911 (0.750–1.106)	0.347

*Note:* Hazard ratios are provided with 95% confidence intervals in parentheses (*p < 0.10, **p < 0.05, ***p < 0.001).

The results of the Gompertz analysis for presence or absence of individual biomarkers are shown in Table 4. The presence of CO, CF/CH, and PH are each associated with significantly higher risks of death. Although the presence of PNBF is also associated with significantly higher risks of death (*p* = 0.012), the 95% confidence interval for the estimate of the effect of the PNBF biomarker covariate spans zero. In addition to not agreeing with the results of the Cox proportional hazards analyses, this result may suggest that the association between PNBF and increased risk of death is a statistical artifact and should not be included in any frailty indices derived from these analyses. Conversely, the presence of OA is associated with significantly lower risks of death. There are no significant associations between risk of death and the presence of LEH, HL, SS, SN, or AM.

**TABLE 4:** Gompertz analysis of presence versus absence of individual biomarkers.

Biomarker	Covariate estimate (95% CI)	LRT	*p*
*Cribra orbitalia* (CO) *n* = 290	0.6287 (0.2362–0.9759)	18.43	< 0.001***
*Cribra femoralis* and/or *humeralis* (CF/CH) *n* = 393	0.8450 (0.4375–1.2038)	32.1	< 0.001***
Porotic hyperostosis (PH) *n* = 313	0.8536 (0.2560–1.3522)	17.086	< 0.001***
Linear enamel hypoplasia (LEH) *n* = 250	0.1689 (−0.1263–0.4375)	1.782	0.182
Harris lines (HL) *n* = 387	0.1560 (−0.1653–0.4463)	1.836	0.175
Stunted stature (SS) *n* = 356	−0.0923 (−1.372–0.8069)	0.076	0.783
Periosteal new bone formation (PNBF) *n* = 371	0.3152 (−0.0366–0.6301)	6.256	0.012**
Osteoarthrosis (OA) *n* = 431	−1.1060 (−1.3329 to (−0.8948))	112.704	< 0.001***
Schmorl's nodes (SN) *n* = 386	−0.1385 (−0.3845–0.0887)	1.848	0.174
Antemortem trauma (AM) *n* = 435	−0.0903 (−0.3493–0.1481)	0.836	0.361

*Note:* Results include maximum likelihood estimates of the effect of the biomarker covariate (with 95% confidence intervals in parentheses) and likelihood ratio tests (LRT) of *H*_*0*_: Effect of biomarker covariate = 0 for the individual biomarkers (*p < 0.10, **p < 0.05, ***p < 0.001).

Based on the results of the preliminary analyses described above, a 4‐biomarker frailty index was constructed. This Milan‐specific index includes CO, CF/CH, PH, and OA. The presence or absence of these biomarkers is consistently and significantly associated with frailty (i.e., in all three of the preliminary analyses). Specifically, the absence of OA and presence of CO, CF/CH, and PH are associated with high frailty (decreased survivorship/increased risk of death), while the presence of OA and absence of CO, CF/CH, and PH reflect low frailty (increased survivorship/decreased risk of death). The index was calculated by assigning one point for each biomarker expression consistent with high frailty—specifically, one point was given for the presence of CO, CF/CH, and PH, and one point for the absence of OA—resulting in a cumulative frailty score ranging from 0 (lowest frailty) to 4 (highest frailty) for each individual. We subsequently applied this index to individuals with all four observable biomarkers. While we could construct 4‐biomarker frailty indices for 275 (of the total 492) individuals, only 269 individuals had both estimated sex and age information.

Binary logistic regressions were employed for the total sample and each period, with estimated sex as the dependent variable and 4‐biomarker index value and point age estimate as independent variables, to assess differences in frailty between females and males within and across historical Milanese periods. Finally, Spearman's correlations were used to evaluate how individual biomarkers (presence/absence) contributed to frailty values by period and sex. As with Kaplan–Meier survival and Cox proportional hazards analyses, these analyses were conducted in SPSS version 29 (IBM Corp 2023).

## 4 Results

Following Yaussy et al. (2024), non‐parametric (Kaplan–Meier), semi‐parametric (Cox proportional hazards), and fully parametric (Gompertz hazards) approaches were used in the preliminary analyses of the individual biomarkers to confirm that each result was accurate and not a statistical artifact. Given the overall consistency of the results produced in those analyses, the performance of the resulting 4‐biomarker frailty index was assessed using only non‐parametric (Kaplan–Meier) and semi‐parametric (Cox proportional hazards) approaches.

The results of Kaplan–Meier analysis of the 4‐biomarker frailty index are shown in Table 5. These results indicate that higher index values are consistently associated with significantly lower survivorship; survivorship steadily decreases with increasing index values. The results of the Cox proportional hazards analysis of the 4‐biomarker frailty index are included in Table 6. In this analysis, individuals with frailty index scores of 0 are the reference group for each comparison. Complementing Kaplan–Meier findings, these results reveal consistent, significant increases in hazards of death with increasing frailty index values.

**TABLE 5:** Kaplan–Meier analysis of 4‐biomarker frailty index.

Index score	Mean age at death (95% CI)	*p*
0	59.395 (56.469–62.320)	< 0.001***
1	42.989 (39.941–46.037)	< 0.001***
2	36.889 (33.016–40.762)	< 0.001***
3	32.063 (25.281–38.844)	< 0.001***
4	24.875 (15.626–34.124)	< 0.001***

*Note:* Mean survival times (mean ages at death) in years are shown with 95% confidence intervals in parentheses (*p < 0.10, **p < 0.05, ***p < 0.001).

**TABLE 6:** Cox proportional hazards analysis of 4‐biomarker frailty index.

Index score	Exp(*β*) (95% CI)	*p*
1	2.098 (1.570–2.805)	< 0.001***
2	3.013 (2.086–4.354)	< 0.001***
3	3.948 (2.298–6.785)	< 0.001***
4	8.601 (3.108–23.802)	< 0.001***

*Note:* Individuals with index scores of 0 are the reference group for each comparison (*p < 0.10, **p < 0.05, ***p < 0.001).

**FIGURE 1:**
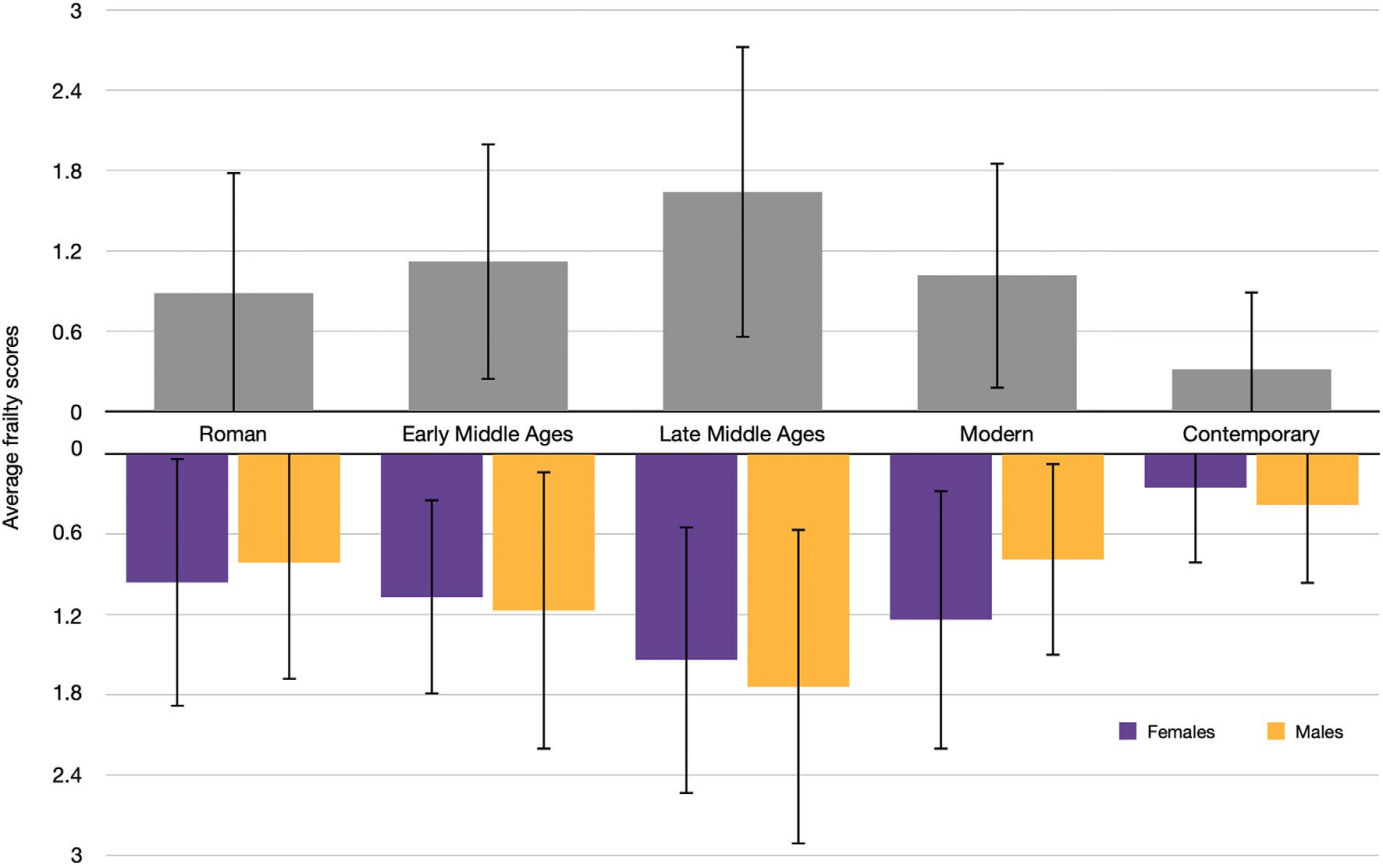
Distribution of average frailty score in the dataset per historical period overall and by estimated sex.

Frailty scores by sex and period are presented in Table 7 and Figure 1. Significant differences in frailty values are reported by period. Notably, significantly higher frailty (1.64 ± 1.1) is observed in individuals from the Late Middle Ages, and significantly lower frailty (0.31 ± 0.58) is observed in individuals from the Contemporary period. Binary logistic regressions (Table 9) yielded no significant differences in frailty between males and females within periods. However, differences in age are observed between females and males in Roman and Contemporary periods; namely, adult males (44.20 ± 11.86 years) lived longer than adult females (37.24 ± 11.34 years) in the Roman period, while Contemporary adult females (50.45 ± 19.66 years) lived significantly longer than Contemporary adult males (45.71 ± 16.24 years).

**TABLE 7:** Frequencies of frailty scores (0–4) by period and estimated sex (Est. Sex).

	Est. Sex	0	1	2	3	4
Roman era	Female (*n* = 23)	8 (34.8%)	10 (43.5%)	3 (13.0%)	2 (8.7%)	0 (0%)
	Male (*n* = 27)	12 (44.4%)	9 (33.3%)	5 (18.5%)	1 (3.7%)	0 (0%)
Early Middle Ages	Female (*n* = 14)	2 (14.3%)	10 (71.4%)	1 (7.1%)	1 (7.1%)	0 (0%)
	Male (*n* = 18)	5 (27.8%)	8 (44.4%)	2 (11.1%)	3 (16.7%)	0 (0%)
Late Middle Ages	Female (*n* = 28)	4 (14.3%)	10 (35.7%)	10 (35.7%)	3 (10.7%)	1 (3.6%)
	Male (*n* = 31)	5 (16.1%)	8 (25.8%)	11 (35.5%)	4 (12.9%)	3 (9.7%)
Modern era	Female (*n* = 17)	4 (23.5%)	7 (41.2%)	4 (23.5%)	2 (11.8%)	0 (0%)
	Male (*n* = 24)	9 (37.5%)	11 (45.8%)	4 (16.7%)	0 (0%)	0 (0%)
Contemporary era	Female (*n* = 48)	39 (81.3%)	6 (12.5%)	3 (6.3%)	0 (0%)	0 (0%)
	Male (*n* = 39)	26 (66.7%)	11 (28.2%)	2 (5.1%)	0 (0%)	0 (0%)

While frailty indices do not reveal significant differences between sexes overall or by period, Spearman's correlations provide greater detail regarding contributing factors of frailty (Table 10). Despite correlations between biomarkers and frailty index values overall and by sex, these patterns do not hold within periods. In the Roman period, for example, PH does not correlate significantly with frailty indices, despite correlations with CO, CF/CH, and OA. In the Early Middle Ages, frailty indices among males and females also do not correlate with PH. Among females, there is also a lack of significant correlation between frailty index values and absence of OA. CO and CF/CH, however, are highly correlated (rho > 0.600) with frailty scores in the Early Middle Ages, as observed in the Roman period individuals. During the Late Middle Ages, frailty values correlate significantly with all biomarkers in males and females, although the correlations between frailty and CO (rho = 0.546, rho = 0.408) and CF/CH (rho = 0.441, rho = 0.540) are notably lower than previous periods. In the Modern individuals, no significant correlations are observed in females between frailty and CO or PH, and males show no significant correlations between frailty and CF/CH or PH. For the Contemporary sample, all significant correlations, aside from OA, are below 0.500. No significant correlation is observed between frailty index values and PH in Contemporary females.

## 5 Discussion

Previous bioarchaeological and paleoepidemiological research on the same Milanese skeletal sample revealed higher mortality risks and reduced survivorship for females, varying across historical periods (Biehler‐Gomez et al. 2024b). Building on these findings, we hypothesized that the frailty index developed in this study, specifically tailored to the Milanese population, would reflect higher cumulative frailty among females. However, results herein demonstrated a more complicated relationship between estimated sex and frailty. Binary logistic regression analysis revealed no significant differences in frailty index values between sexes (Table 9). While differences in average frailty indices were observed between males and females in the combined sample (all individuals) and within specific historical periods—sometimes favoring males and at other times females (Table 8)—these differences lacked statistical significance. This finding suggests that, despite documented differences in mortality risks and survival between sexes over time, there is no clear evidence of sex‐based differences in cumulative frailty, and the relationship between morbidity and mortality in this urban population is more complex than we hypothesized.

**TABLE 8:** Skeletal frailty and point age estimate distributions by sex and period.

	Female			Male		
	*n*	4‐biomarker frailty index	Estimated point ages	*n*	4‐biomarker frailty index	Estimated point ages
		Average (SD)	Average (SD)		Average (SD)	Average (SD)
Roman era	23	0.96 (0.93)	37.24 (11.34)	27	0.81 (0.88)	44.20 (11.86)
Early Middle Ages	14	1.07 (0.73)	40.86 (11.96)	18	1.17 (1.04)	36.75 (14.68)
Late Middle Ages	28	1.54 (1.00)	40.59 (16.15)	31	1.74 (1.18)	38.97 (13.52)
Modern era	17	1.24 (0.97)	41.82 (13.75)	24	0.79 (0.72)	44.98 (11.98)
Contemporary era	48	0.25 (0.57)	68.39 (14.86)	39	0.38 (0.59)	56.70 (18.24)
All	130	0.87 (0.95)	50.45 (19.66)	139	0.94 (1.01)	45.71 (16.24)

However, in both the Roman and Contemporary periods, frailty scores varied significantly by age‐at‐death (Table 9). In the Roman period, previous studies evidenced lower survivorship and higher mortality risks for females (Biehler‐Gomez et al. 2024b). The current findings, however, suggest that this disparity in mortality was not mirrored by increased morbidity, as females did not exhibit significantly higher frailty index values. This raises the possibility that external factors, such as pregnancy and childbirth, significantly contributed to female mortality in this period, as these events are well documented as high‐risk in Roman society (Challet 2017; Rouselle 1990, 319).

**TABLE 9:** Binary logistic regression comparing index scores and age (independent variables) with estimated sex (dependent variable) overall and by period.

	B	S.E.	Wald	df	Sig.	Exp(*β*)	Exp(*β*) (95% CI)	
							Lower	Upper
All								
4‐biomarker frailty index	−0.100	0.150	0.446	1	0.504	0.904	0.673	1.215
Point age	−0.018	0.008	4.630	1	0.031**	0.983	0.967	0.998
Constant	1.007	0.498	4.078	1	0.043**	2.736		
Roman era								
4‐biomarker frailty index	0.113	0.364	0.096	1	0.757	1.119	0.548	2.286
Point age	0.057	0.029	3.774	1	0.052*	1.059	0.999	1.122
Constant	−2.267	1.378	2.705	1	0.100	0.104		
Early Middle Ages								
4‐biomarker frailty index	−0.161	0.520	0.096	1	0.756	0.851	0.307	2.356
Point age	−0.030	0.035	0.742	1	0.389	0.970	0.906	1.039
Constant	1.604	1.826	0.772	1	0.380	4.975		
Late Middle Ages								
4‐biomarker frailty index	0.160	0.257	0.389	1	0.533	1.174	0.710	1.941
Point age	−0.004	0.019	0.041	1	0.839	0.996	0.960	1.034
Constant	−0.007	1.006	0.000	1	0.994	0.993		
Modern era								
4‐biomarker frailty index	−0.651	0.452	2.072	1	0.150	0.522	0.215	1.265
Point age	0.000	0.029	0.000	1	0.994	1.000	0.945	1.059
Constant	0.988	1.564	0.399	1	0.528	2.686		
Contemporary era								
4‐biomarker frailty index	−0.249	0.471	0.280	1	0.597	0.780	0.310	1.961
Point age	−0.046	0.017	7.850	1	0.005**	0.955	0.924	0.986
Constant	2.808	1.162	5.843	1	0.016**	16.569		

*Note:* The lack of significance in most periods. (*p < 0.10, **p < 0.05, ***p < 0.001).

Abbreviations: B, regression coefficient; CI, confidence interval; df, degrees of freedom; SE, standard error.

Frailty index values exhibited notable variation across historical periods, following a general increase in frailty from the Roman era (0.96 for females, 0.81 for males) to the Late Middle Ages, where they peaked (1.54 for females, 1.74 for males). This was followed by a sharp decline in frailty values, reaching their lowest levels in the Contemporary period (0.25 for females, 0.38 for males) (Figure 1, Table 8). These patterns were consistent across sexes, reflecting similar trends for both males and females. The results of the frailty index will be examined by period.

### 5.1 Roman Era

Frailty scores in the Roman sample were predominantly distributed between the two lowest scores, 0 and 1, with no individuals, male or female, reaching a score of 4 (Table 7). While females exhibited slightly higher average frailty compared to males (0.96 and 0.81, respectively—Table 8), this difference was not statistically significant. However, males had a significantly higher average age‐at‐death than females (44.20 years vs. 37.34 years, respectively—Tables 8 and 9). This observation is supported by previous Kaplan–Meier survival analyses, which revealed significantly lower female survivorship during this period (Biehler‐Gomez et al. 2024b). Within this sample, CO, CF/CH, and OA were significantly correlated with frailty index values, whereas this was not the case for PH (Table 10).

**TABLE 10:** Results for 4‐biomarker frailty index values in all individuals, female individuals, and male individuals overall and within period compared with high (“1”) frailty biomarkers.

			CO	CF/CH	PH	OA
All individuals	All (*n* = 275)	rho	0.590**	0.545**	0.452**	0.718**
		*p*	0.000	0.000	0.000	0.000
	Females (*n* = 134)	rho	0.533**	0.611**	0.390**	0.754**
		*p*	0.000	0.000	0.000	0.000
	Males (*n* = 141)	rho	0.642**	0.487**	0.501**	0.693**
		*p*	0.000	0.000	0.000	0.000
Roman era	All (*n* = 51)	rho	0.579**	0.622**	0.169	0.714**
		*p*	0.000	0.000	0.237	0.000
	Females (*n* = 24)	rho	0.515*	0.757**	NA	0.878**
		*p*	0.010	0.000	—	0.000
	Males (*n* = 27)	rho	0.686**	0.512**	0.253	0.614**
		*p*	0.000	0.006	0.203	0.001
Early Middle Ages	All (*n* = 34)	rho	0.665**	0.620**	0.428*	0.575**
		*p*	0.000	0.000	0.012	0.000
	Females (*n* = 16)	rho	0.639**	0.639**	0.388	0.488
		*p*	0.008	0.008	0.137	0.055
	Males (*n* = 18)	rho	0.698**	0.610**	0.452	0.641**
		*p*	0.001	0.007	0.060	0.004
Late Middle Ages	All (*n* = 61)	rho	0.495**	0.451**	0.631**	0.659**
		*p*	0.000	0.000	0.000	0.000
	Females (*n* = 29)	rho	0.546**	0.408*	0.541**	0.572**
		*p*	0.002	0.028	0.002	0.001
	Males (*n* = 32)	rho	0.441*	0.540**	0.678**	0.758**
		*p*	0.012	0.001	0.000	0.000
Modern era	All (*n* = 42)	rho	0.455**	0.520**	0.377*	0.621**
		*p*	0.002	0.000	0.014	0.000
	Females (*n* = 17)	rho	0.244	0.726**	0.463	0.637**
		*p*	0.346	0.001	0.061	0.006
	Males (*n* = 25)	rho	0.586**	NA	0.290	0.687**
		*p*	0.002	—	0.160	0.000
Contemporary era	All (*n* = 87)	rho	0.377**	0.472**	0.232*	0.870**
		*p*	0.000	0.000	0.031	0.000
	Females (*n* = 48)	rho	0.349*	0.498**	NA	0.995**
		*p*	0.015	0.000	—	0.000
	Males (*n* = 39)	rho	0.373*	0.463**	0.323*	0.750**
		*p*	0.019	0.003	0.045	0.000

Abbreviations: CF/CH, *cribra femoralis/humeralis* (presence); CO, *cribra orbitalia* (presence); *n*, number of cases; NA, not applicable; OA, osteoarthrosis (absence); *p*, *p* value; PH, porotic hyperostosis (presence); Rho, correlation coefficient.

*p < 0.10, **p < 0.05, ***p < 0.001.

These findings suggest that although Roman period females experienced reduced longevity compared to their male counterparts, their frailty index values indicate comparable levels of morbidity between the sexes. As previously mentioned, this disparity in mortality may be attributable to external factors affecting female frailty, such as the risks associated with pregnancy and childbirth (Fojas 2022; World Health Organization 2023), rather than differences in cumulative morbidity. As historical references (Cicerone, *Epistulae ad familiars*, VI, 18.2; Plinio il Giovane, *Epistulae*, IV, 21; Plutarco, *Vita di Silla*, 33) and epigraphical Roman sources (Challet 2017; Rouselle 1990, 319) attest, maternal mortality was a major cause of shortened female lifespans during this period. Collectively, increased mortality and comparable levels of frailty with male counterparts testify to a historic period in Milan where cultural, societal, and political circumstances severely and disproportionately affected female longevity and morbidity.

### 5.2 Early Middle Ages

As with Roman period individuals, frailty scores in the Early Middle Ages were also primarily concentrated between 0 and 1. However, the frequency of higher scores, particularly score 3, increased in this period (Table 7). This shift resulted in slightly higher average frailty index values compared to the Roman period, although the increase was not statistically significant. Males demonstrated marginally higher frailty scores than females (1.17 and 1.07, respectively—Table 8), but this difference also lacked statistical significance. Similarly, average age‐at‐death was comparable between sexes, with no statistically significant difference observed (40.86 years for females and 36.75 years for males—Table 8). As in the Roman period, PH did not significantly correlate with the 4‐biomarker frailty index, while other biomarkers demonstrated significant associations (Table 10).

Albeit not a significant increase in frailty between Roman and Early Middle Ages, the general change in frailty, notably in the higher frequency of elevated frailty scores, may reflect political, social, economic, and cultural changes associated with the transformation of the Western Roman Empire. With the geopolitical decline of the Western Roman Empire, communities in west and northwestern continental Europe suffered political instability and acute demographic crisis, exacerbated by inclusion policies, higher taxes, and trade collapse (Harper 2017). Living conditions thus slowly worsened throughout the period known as Late Antiquity (Brown 1998), which may explain the frailty scores observed between these two historical periods. In fact, in the Early Middle Ages, Milan was subjected to successive invasions, wars, epidemics, famines, and social instability, and even destroyed in 539 ce (Gasparri and La Rocca 2012; Le Glay et al. 2011; Provero and Vallerani 2016; da Silva 2016; Tsiamis et al. 2011).

### 5.3 Late Middle Ages

The Late Middle Ages were marked by an increase in frailty scores, with the highest frequencies observed at scores 1 and 2. Notably, this was the only period in which individuals exhibited maximal frailty scores, 4 (Table 7). The average frailty index for this period was significantly higher than earlier periods, with scores of 1.54 for females and 1.74 for males (*p* = 0.001). Age‐at‐death was similar between sexes (40.59 years for females and 38.97 years for males—Table 8), with no statistically significant differences. All four biomarkers significantly contributed to the frailty index during this period (Table 10).

It is interesting to note that individuals in the Late Middle Ages exhibited the highest frailty scores among the five periods considered in this study, rather than those from the Early Middle Ages, a period that has been traditionally, though problematically, portrayed in popular discourse as the “dark ages” of European history (Mommsen 1942). Throughout the Middle Ages, poverty was a widespread and persistent condition. Many individuals lived in precarious circumstances, often deprived of food, stable housing, and personal freedom (Mollat 1982). Various categories of poverty were recognized at the time, including the *laboriosus* and the *indigens*, workers who lived in a state of general deprivation and vulnerability. Others included the *famelicus* (the starving), the *nudus* (those without clothing), the sick, individuals with mental impairments, the elderly, widows, orphans, pregnant women, the *captivus* (those deprived of their liberty), the exiled, the *verecundus* (those who had fallen into disgrace and lost their social standing and much of their wealth), the *spontaneus* (the voluntary poor), migrants, and pilgrims (who were often temporarily or voluntarily impoverished) (Fornasiero 2023; Mollat 1982). During the Late Middle Ages, three major scourges struck with devastating intensity: war, famine, and plague, often accompanied by increasingly frequent and severe natural disasters. These events had a dramatic impact on the population, leading to a significant demographic decline (Bellosta 2023), major economic transformations, and a severe nutritional crisis caused by food shortages (Albini 2016). Epidemics, in particular, thrived in overcrowded living conditions with poor hygiene, exacerbated by the constant movement of armies, which acted as powerful vectors of contagion. Although no social class was entirely spared, the poorest segments of society bore the heaviest burden, lacking the resources necessary to flee urban centers or access effective medical care (Albini 2016).

The present findings align with previous bioarchaeological studies using a cumulative phenotype approach to the analysis of frailty in Milan (Petrosino et al. 2025) as well as with a growing body of literature challenging this historical stigma and shedding light on the nuanced living conditions of these periods (e.g., Steckel 2004; Tilley and Cave 2023).

### 5.4 Modern Era

Frailty index scores in the Modern era were more evenly distributed among scores 0, 1, and 2 (Table 7), resulting in a significant decrease in frailty index averages compared to the Late Middle Ages (*p* = 0.021). Although females exhibited higher average frailty scores than males (1.24 and 0.79, respectively), this difference was not statistically significant. Similarly, average age‐at‐death was lower for females (41.82 years) than for males (44.98 years), though the difference was not significant (Table 8). Previous Kaplan–Meier analyses on Milanese males and females, however, revealed significantly lower survivorship for females during this period (Biehler‐Gomez et al. 2024b). While the Modern era encompasses ideological and social revolutions (e.g., Renaissance, Age of Enlightenment), frailty scores during the Modern era compare with those of the Roman period and Early Middle Ages.

Interestingly, although all four biomarkers were significantly associated with frailty scores, this varied by sex: CO (presence) and OA (absence) significantly contributed to male frailty scores, whereas female frailty was affected by CF/CH (presence) and OA (absence) (Table 10). This divergence may reflect early lifetime disparities between sexes, as the window of formation of these cribriotic lesions slightly differs due to differences in marrow conversion timing depending on the skeletal area considered. In fact, CF/CH lesions can form earlier than CO lesions (Brickley 2018), suggesting that Modern females may have experienced earlier exposure to environmental stressors than Modern males. While likely one of many explanations, this difference may be linked to gendered labor practices during the Modern period. With the intensification of the textile industry in Milan (Battistini 2009; Bellavitis 2018), women, in particular, started as young girls in the needle trade for textile and especially silk‐manufacturing (Caracausi 2014; Quataert 1985). This early exposure to stressful factory labor and working conditions for females may partially explain the differential biomarker contributions to the frailty index in the Modern period.

### 5.5 Contemporary Era

The Contemporary period exhibited the lowest frailty scores across the entire sample, with the large majority of scores concentrated at 0 (Table 7). This resulted in significantly lower average frailty index scores compared to all preceding periods (*p* < 0.001). Although differences in frailty scores between sexes were not statistically significant (0.25 for females and 0.38 for males), binary logistic regressions revealed males dying significantly earlier than females (average of 68.39 years for females and 56.70 years for males—Tables 8 and 9), consistent with previous findings (Biehler‐Gomez et al. 2024b). In this sample, all biomarkers contributed to male frailty scores, while PH had no significant effect on female frailty (Table 10).

The marked decline in frailty scores during the Contemporary period reflects significant improvements in living conditions over the past two centuries, aligning with bioarchaeological studies and current epidemiological trends (e.g., Austad and Fischer 2016; Hens et al. 2019; Zarulli et al. 2018). The lifespan and frailty data observed in the Contemporary Milanese samples, with females exhibiting older average ages‐at‐death (mortality) and showing skeletal frailty levels (morbidity) comparable to males, demonstrate a morbidity‐mortality paradox (di Lego et al. 2022). Today, women live an average of 4.4 years longer than men, with life expectancies of 74.2 and 69.8 years, respectively (World Health Organization 2019). While surviving longer, over their lifetimes women also experience more illnesses and greater morbidity than their male contemporaries along with the additional stresses of childbearing and caregiving. Jointly, these two trends produce a male–female morbidity‐mortality paradox across current populations (di Lego et al. 2022; World Health Organization 2019). A major contributing factor to earlier male mortality across populations today is their comparatively low use of available healthcare services relative to contemporary women (see World Health Organization 2019). These results indicate that, for the first time in the historical sequence analyzed, females outlived males despite comparable skeletal frailty levels, aligning with modern epidemiological patterns and potentially marking the emergence of the morbidity‐mortality paradox in this population.

From Roman to Modern periods, lack of frailty differences speaks to cultural buffering of males over females, results observed in other bioarchaeological frailty research (Yaussy et al. 2024). In the Roman period, especially, decreased longevity and comparable frailty in females reflect the measurable costs in mortality and morbidity associated with being a female under Roman imperial rule; the role and purpose of females were predicated on their relationship to male relatives, with the *paterfamilias* exerting authority over all aspects of domestic life, including access to resources, healthcare, and social and legal decisions (Franciosi 1992; Guarino 1992, 225–244; Lee 1946, 64–68). Preferential male buffering continued until the Contemporary period in Milan, when we observe firsthand the female–male morbidity–mortality paradox. Increased longevity in females and comparable frailty between sexes demonstrate how contemporary actions to dismantle patriarchal political and social systems, in favor of gender equality, directly contribute to improvements in female survivability and stress. In particular, the Contemporary females and males represent individuals who grew up and lived in an Italy when women could vote (Legislative Decree of February 1st, 1945), pursue public professions (Law 66/63, 1963), receive equal opportunities in the work force (Law 903/77, Article 1, 1977), and make reproductive decisions (Law 194/78, 1978). Unlike their female predecessors, whose civil and human rights were not codified into law, Contemporary Milanese females benefited biologically from present‐day legal protections, medical care, and social services, which resulted in not only longer but less frail lives.

As with other indexical measures of skeletal frailty—the Health Index (Steckel et al. 2002), Skeletal Frailty Index (Marklein and Crews 2017), and Biological Index of Frailty (Zedda et al. 2021)—this hazards‐based cumulative approach to frailty has limitations that should be considered when applying this methodology to other past populations, namely sample representation. Of the 492 individuals originally included in this study, only 269 (55%) individuals had observable all four biomarkers associated with decreased survival and increased mortality. Sample representation and comparability are critical issues when considering whether an indexical approach is informative and which indexical approach is appropriate for evaluating frailty. Additionally, as Petrosino et al. (2025) outlined, future methodologies for frailty may not only include new biomarkers but also incorporate aspects of lesion processes and development, rather than binary or scalar categories. These operationalizations of frailty may also improve from considerations of frailty outcomes in the living and early life stressors.

## 6 Conclusion

The application of a Milanese‐specific frailty index facilitated a diachronic comparison of frailty from Roman to Contemporary periods. In addition to observing changes in frailty over 2000 years of Milanese history, the 4‐biomarker frailty index also quantified cumulative measures of stress between estimated adult females and males. Overall findings between females and males presented no significant differences in frailty; results echoed within the five discrete time periods. However, significant differences in age distribution between sexes were observed within Roman (older males) and Contemporary (older females) samples. These results attest to the presence of male cultural buffering in historical Milan, up until the Contemporary period. More generally, this study highlights the utility of using context‐specific frailty indices that leverage the data available for a particular region or time period and that might best reflect environmentally‐ and culturally‐mediated exposures to conditions that shape health (see also, Biehler‐Gomez, Yaussy, et al. 2025; Yaussy et al. 2024).

## Author Contributions

**Lucie Biehler‐Gomez:** conceptualization, project administration, investigation, writing – original draft, writing – review and editing, formal analysis, methodology. **Kathryn E. Marklein:** conceptualization, methodology, investigation, writing – original draft, writing – review and editing, formal analysis. **Samantha L. Yaussy:** conceptualization, methodology, formal analysis, writing – original draft, writing – review and editing, investigation. **Douglas E. Crews:** writing – original draft, writing – review and editing, investigation. **Sharon N. DeWitte:** writing – original draft, writing – review and editing, investigation. **Cristina Cattaneo:** resources, data curation.

## Conflicts of Interest

The authors declare no conflicts of interest.

## Data Availability

The data that support the findings of this study are available from the corresponding author upon reasonable request.

## Endnotes

^1^
For instance, through press articles, e.g., https://www.ilgiorno.it/milano/cronaca/differenze‐di‐genere‐e‐longevita‐tra‐rischi‐diritti‐e‐lavori‐usuranti‐cosi‐e‐cambiata‐la‐vita‐delle‐donne‐a89f8a4a, https://www.cronachediscienza.it/2024/10/16/un‐nuovo‐studio‐riscrive‐la‐storia‐della‐mortalita‐e‐della‐sopravvivenza‐femminile‐a‐milano‐negli‐ultimi‐2mila‐anni/, https://storiearcheostorie.com/2024/10/14/studi‐secoli‐bui‐di‐certo‐non‐a‐milano‐qui‐le‐donne‐nel‐medioevo‐vivevano‐piu‐a‐lungo‐rispetto‐allepoca‐romana/.
